# Adrenocortical Carcinoma Presenting With Concurrent Cushing Syndrome and Primary Hyperaldosteronism

**DOI:** 10.1016/j.aed.2026.02.003

**Published:** 2026-02-18

**Authors:** Naseem Eisa

**Affiliations:** Community Health Partners, Fresno, California

**Keywords:** adrenocortical carcinoma, Cushing's syndrome, hyperaldosteronism, mitotane, Weiss score, primary aldosteronism

## Abstract

**Background/Objective:**

Adrenocortical carcinoma (ACC) is a rare and aggressive endocrine malignancy, with approximately half of cases demonstrating hormonal hypersecretion. Cortisol excess resulting in Cushing syndrome is the most common functional presentation. In contrast, aldosterone-producing ACC is distinctly uncommon, and concurrent secretion of both cortisol and aldosterone by a single tumor is rare. We describe a case of dual hormone–secreting ACC and review its diagnostic and therapeutic implications.

**Case Report:**

A 64-year-old woman with long-standing treatment-resistant hypertension and poorly controlled type 2 diabetes mellitus presented with progressive weight gain, proximal muscle weakness, and worsening metabolic control. Biochemical evaluation demonstrated adrenocorticotropic hormone-independent hypercortisolism together with primary hyperaldosteronism. Computed tomography revealed a heterogeneous 4.3-cm left adrenal mass with indeterminate imaging characteristics suspicious for malignancy. Following preoperative endocrine optimization, including correction of hypokalemia and management of hypercortisolism, the patient underwent successful en bloc left adrenalectomy. Histopathologic examination confirmed adrenocortical carcinoma with a Weiss score of 6 and a Ki-67 proliferation index of approximately 25%, consistent with high-risk disease. Final staging was ENSAT stage II. Postoperatively, the patient experienced marked improvement in blood pressure control and glycemic parameters. Given high-risk pathologic features, adjuvant mitotane therapy was initiated.

**Discussion:**

Dual cortisol- and aldosterone-secreting ACC is rare, with only a small number of cases reported in the literature. This phenotype likely reflects disordered steroidogenesis associated with underlying molecular abnormalities.

**Conclusion:**

Comprehensive hormonal evaluation of adrenal masses is essential, as early recognition and multidisciplinary management of dual-secreting ACC can lead to improved clinical outcomes.

**Highlights**

• Dual secretion of cortisol and aldosterone by adrenocortical carcinoma (ACC) is exceptionally rare, with only 14 prior cases reported in the literature from 2004 to 2025
• Comprehensive hormonal evaluation of all adrenal masses is essential, as dual hormone secretion may be missed if testing is limited to the clinically dominant syndrome
• Preoperative optimization in dual-secreting ACC requires correction of hypokalemia and management of both hypercortisolism and hyperaldosteronism to minimize perioperative cardiovascular risks
• Postoperative adrenal insufficiency should be anticipated in patients with cortisol-secreting ACC due to chronic suppression of the hypothalamic-pituitary-adrenal axis
• High-risk pathologic features (Ki-67 > 10%, hormone secretion) warrant consideration of adjuvant mitotane therapy following complete surgical resection

**Clinical Relevance**

This case highlights the diagnostic challenge of dual hormone secretion in adrenocortical carcinoma and demonstrates that a comprehensive, multidisciplinary approach—integrating biochemical, radiological, surgical, and pathologic expertise—is essential for achieving hormonal remission and optimizing patient outcomes.

## Introduction

Adrenocortical carcinoma (ACC) represents a rare and aggressive endocrine neoplasm, with an annual incidence of approximately 1 to 2 cases per million individuals.^1^ A significant proportion of these tumors, up to 60%, are functionally active, precipitating various syndromes of hormone excess.^1^ The most common of these is Cushing's syndrome, driven by autonomous cortisol production, often accompanied by hyperandrogenism.^2^ In stark contrast, primary hyperaldosteronism is an infrequent presentation of ACC, identified in fewer than 2% of patients.^3^ The synchronous hypersecretion of both cortisol and aldosterone from a single adrenocortical tumor is a rare clinical finding,^4^ with only 14 cases described in the medical literature from 2004 to 2025 (Table 1).^5^, ^6^, ^7^, ^8^, ^9^, ^10^, ^11^ These rare presentations pose considerable diagnostic and therapeutic dilemmas, mandating a meticulous and comprehensive endocrine workup and a coordinated, multidisciplinary management strategy.^4^ This case report contributes to the existing body of knowledge on dual-secreting ACC and reinforces the critical need for exhaustive endocrine evaluation in any patient presenting with an adrenal mass.

**Table 1:** Literature Review of Dual Aldosterone-Cortisol Secreting Adrenocortical Carcinoma Cases (2004-2025)

#	Author	Year	Age/Sex	Size (cm)	Hormones secreted	Ki-67	Weiss	Follow-up (mo)	Outcome
1	Kurtulmus et al^5^	2004	26/F	NR	Aldo + Subclinical cortisol	NR	NR	36	NED
2	Barzon et al^6^	2005	NR/F	NR	Aldo → Cortisol (shift)	NR	NR	NR	Recurrence
3	Peppa et al^7^	2009	59/F	6.5	Cortisol + Aldo + Androgens	25%	NR	6	Liver mets
4	Beom et al.^12^	2011	72/M	NR	Cortisol + Aldo	NR	NR	<1	Death
5	Choi et al.^13^	2011	70/F	NR	Cortisol + Aldo + Androgens	NR	NR	NR	On mitotane
6	Ha et al.^14^	2012	77/F	NR	Cortisol + Aldo	NR	NR	NR	On HC
7	Chowdhury et al.^15^	2014	37/F	6.0	Aldo + Cortisol	NR	NR	11	Death
8	Lazaro et al^8^	2018	18/M	4.7	Aldo + Cortisol + Estradiol	5-10%	4	NR	NED
9	Punni et al.^16^	2020	59/F	6.5	Aldo + Cortisol	NR	NR	NR	On follow-up
10	Rosenberg et al^4^	2023	63/M	3.5	Aldo + Cortisol	68%	6	9	NED
11	Aliño et al^9^	2024	44/F	23.0	Cortisol + Aldo	>20%	4	NR	On adjuvant RT
12	Woo et al^10^	2024	57/F	7.0	Cortisol + Aldo + NMN	NR	NR	NR	NR
13	Ezzahra et al^11^	2025	46/M	9.0	Aldo + Cortisol	NR	3	NR	BP normalized
14	Musacchio et al.^17^	2025	55/F	8.9	Cortisol + Aldo (evolved)	60%	NR	NR	Metastatic
15	Present case	2025	64/F	4.3	Cortisol + Aldo	25%	6	6	NED

Abbreviations: Aldo, aldosterone; BP, blood pressure; F, female; HC, hydrocortisone; M, male; mets, metastases; mo, months; NED, no evidence of disease; NMN, normetanephrine; NR, not reported; RT, radiotherapy.

Table 1 summarizes all reported cases of dual aldosterone- and cortisol-secreting adrenocortical carcinoma in the literature from 2004 to 2025. A total of 15 cases have been identified, including the present case. The cases demonstrate a female predominance (11/15, 73%), a wide age range (18-77 y), and variable tumor sizes (3.5-23.0 cm). Ki-67 proliferation indices, when reported, ranged from 5% to 68%, and Weiss scores ranged from 3 to 6. Some cases exhibited triple hormone secretion with concurrent androgen, estradiol, or catecholamine production. Follow-up duration ranged from less than 1 mo to 36 mo where reported. Outcomes varied from no evidence of disease to metastatic progression and death, reflecting the heterogeneous natural history of this rare tumor type.

## Case Presentation

A 64-year-old female was referred to our endocrinology service for the management of refractory hypertension and poorly controlled type 2 diabetes. Her past medical history was significant for a surgically repaired 7.0-cm abdominal aortic aneurysm, chronic hypertension, hyperlipidemia, and a 40-pack-year smoking history. Her blood pressure remained elevated, with an average reading of 178/96 mmHg, despite a regimen of 5 antihypertensive agents (lisinopril, amlodipine, carvedilol, chlorthalidone, and spironolactone). Her glycemic control was similarly suboptimal, with a hemoglobin A1C of 7.9% (62.8 mmol/mol) on a combination of metformin, empagliflozin, tirzepatide, and pioglitazone.

On physical assessment, the patient exhibited classic signs of hypercortisolism, including central obesity (body mass index, 32 kg/m^2^), facial plethora and rounding, dorsocervical and supraclavicular fat accumulation, and proximal muscle weakness.^2^ No evidence of virilization was noted.

### Diagnostic Assessment

An extensive endocrine investigation was undertaken to characterize the patient's hormonal status (Table 2). The biochemical results were conclusive for 2 concurrent yet distinct hormonal syndromes: adrenocorticotropic hormone (ACTH)-independent hypercortisolism and primary hyperaldosteronism.^18^^,^^19^ The diagnosis of adrenal Cushing's syndrome was established by the finding of a suppressed plasma ACTH level in the presence of elevated cortisol production, confirmed by a postdexamethasone suppression cortisol level of 5.3 μg/dL (146 nmol/L) with an adequate serum dexamethasone level of 245 ng/dL.^18^ The diagnosis of primary hyperaldosteronism was confirmed after withdrawal of interfering medications. Spironolactone was discontinued for 6 weeks and lisinopril was held for 2 weeks prior to testing. The resulting aldosterone-to-renin ratio was 189, calculated from a plasma aldosterone of 17 ng/dL and a suppressed plasma renin activity of 0.09 ng/mL/h.^19^ Serum DHEA-S was markedly elevated at 750 μg/dL (normal range for age <150 μg/dL), further increasing suspicion for ACC. A concurrent pheochromocytoma was ruled out by normal levels of plasma free metanephrines and 24-hour urinary catecholamines.^20^

**Table 2:** Hormonal and Biochemical Evaluation Results

Test	Value (Conventional and SI Units)	Normal range
Serum potassium	2.9 mmol/L	3.5-5.1 mmol/L
Serum albumin	4.2 g/dL (42 g/L)	3.5-5.0 g/dL (35-50 g/L)
Hemoglobin A1C	7.9% (62.8 mmol/mol)	<5.7% (<39 mmol/mol)
1-mg overnight DST (serum cortisol)	5.3 μg/dL (146 nmol/L)	<1.8 μg/dL (<50 nmol/L)
Serum dexamethasone level	245 ng/dL (6.3 nmol/L)	>200 ng/dL (>5.1 nmol/L)
Plasma ACTH	<5 pg/mL (<1.1 pmol/L)	7.2-63.3 pg/mL (1.6-13.9 pmol/L)
24-h urinary free cortisol	312 μg/24 h (861 nmol/24 h)	<50 μg/24h (<138 nmol/24h)
Plasma aldosterone concentration	17 ng/dL (0.47 nmol/L) upright	4-31 ng/dL (0.11-0.86 nmol/L) upright
Plasma renin activity	0.09 ng/mL/h upright	0.5-4.0 ng/mL/h upright
Aldosterone-to-renin ratio	189	<30
Serum DHEA-S	750 μg/dL (20.3 μmol/L)	<150 μg/dL (<4.1 μmol/L)
Plasma free metanephrine	0.18 nmol/L	<0.50 nmol/L
Plasma free normetanephrine	0.42 nmol/L	<0.90 nmol/L
24-h urine metanephrines	148 μg/24 h (762 nmol/24 h)	<400 μg/24h (<2060 nmol/24 h)
24-h urine catecholamines	85 μg/24 h (503 nmol/24 h)	<200 μg/24h (<1182 nmol/24 h)

Abbreviations: ACTH, adrenocorticotropic hormone; ARR, aldosterone-to-renin ratio; DHEA-S, dehydroepiandrosterone sulfate; DST, dexamethasone suppression test; SI, International System of Units.

Table 2 presents the comprehensive hormonal and biochemical evaluation performed during the diagnostic workup. The results demonstrate ACTH-independent hypercortisolism (suppressed ACTH with elevated urinary free cortisol and non-suppressed cortisol after dexamethasone), primary hyperaldosteronism (elevated aldosterone with suppressed renin activity yielding an ARR of 189), and markedly elevated DHEA-S consistent with adrenocortical carcinoma. Pheochromocytoma was excluded by normal plasma free metanephrines and urinary catecholamines.

Following the biochemical confirmation of adrenal hyperfunction, an abdominal computed tomography scan was performed to localize the source. The scan revealed a 4.3 × 3.6 cm mass in the left adrenal gland (Fig. 1). The adrenal mass displayed heterogeneous enhancement and coarse calcifications. The lesion's attenuation values (25 HU pre-contrast, 50 HU arteriovenous, 37 HU delayed) resulted in an absolute washout of 52% and a relative washout of 26%, characteristics that are indeterminate for a benign adenoma.^1^

**Fig. 1:**
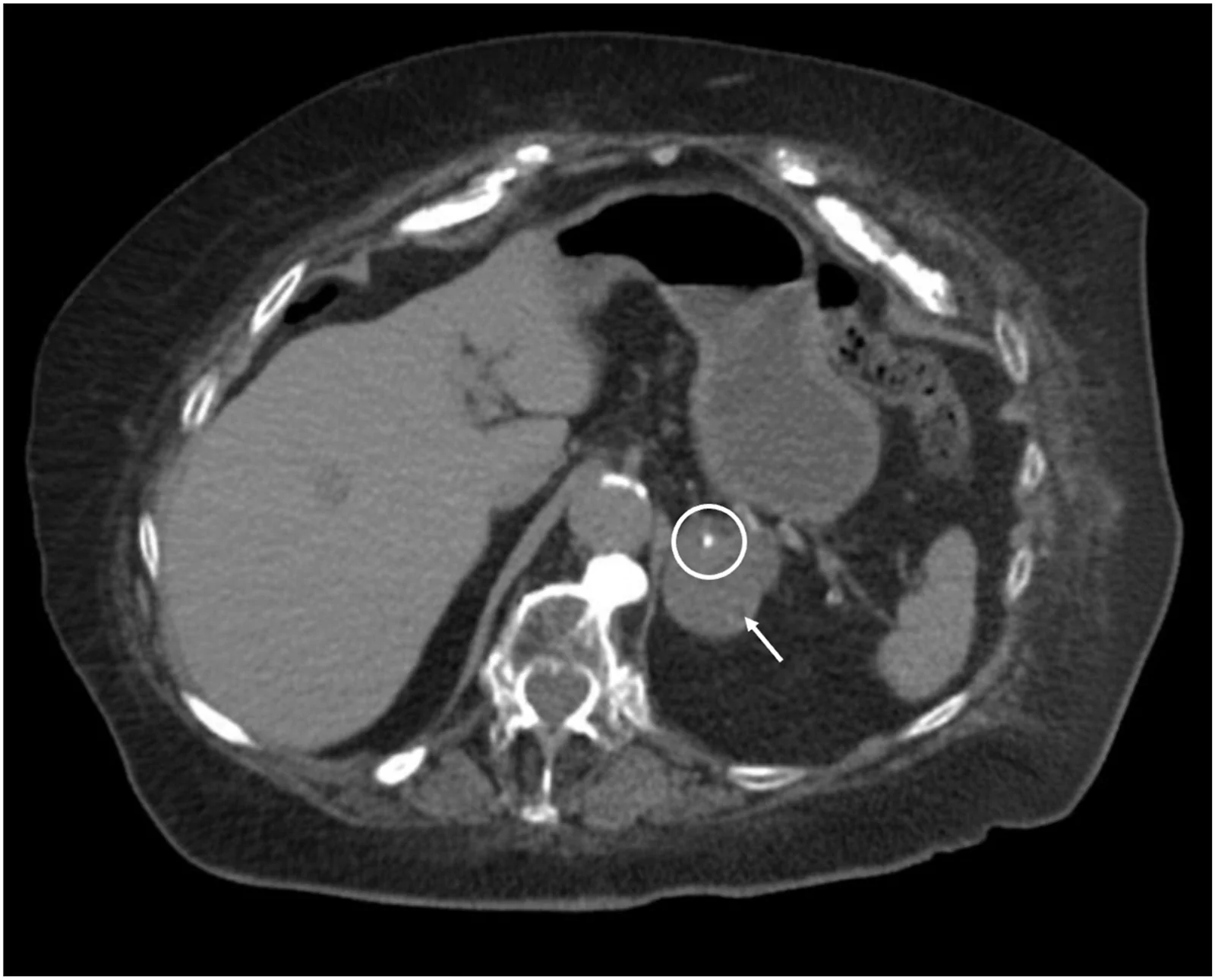
Axial contrast-enhanced computed tomography of the abdomen shows a heterogeneous *left* adrenal mass (arrow) measuring approximately 4.3 × 3.6 cm. The lesion demonstrates intermediate precontrast attenuation (25 Hounsfield units), heterogeneous enhancement, and indeterminate washout characteristics, with coarse internal calcification (circled), findings suspicious for malignant adrenal neoplasm. The *right* adrenal gland is unremarkable, and no radiographic evidence of regional lymphadenopathy or distant metastases is identified.

Based on the constellation of indeterminate imaging, large tumor size, and dual hormonal activity, a surgical approach was recommended.^1^ The patient's profound hypokalemia (2.9 mmol/L) was aggressively managed preoperatively with both intravenous and oral potassium chloride, restoring a safe serum level of 4.0 mmol/L.^19^ Her blood pressure was further managed with the mineralocorticoid receptor antagonist eplerenone.^19^ Subsequently, she underwent a successful en bloc resection of the left adrenal gland and tumor, performed by a surgical oncologist with expertise in adrenal surgery.^1^

The resected specimen revealed a large, well-encapsulated, lobulated tumor surrounded by periadrenal adipose tissue with focal areas of hemorrhage (Fig. 2 *A*). Histologic examination confirmed features of malignancy, including vascular invasion with tumor cells within a blood vessel lumen (Fig. 2 *B*) and sheets of pleomorphic cells with high nuclear grade, prominent nucleoli, abundant eosinophilic cytoplasm, and a mitotic rate greater than 10 per 50 high-power fields (Fig. 2 *C*). These findings yielded a Weiss score of 6, confirming the diagnosis of ACC.^21^

**Fig. 2:**
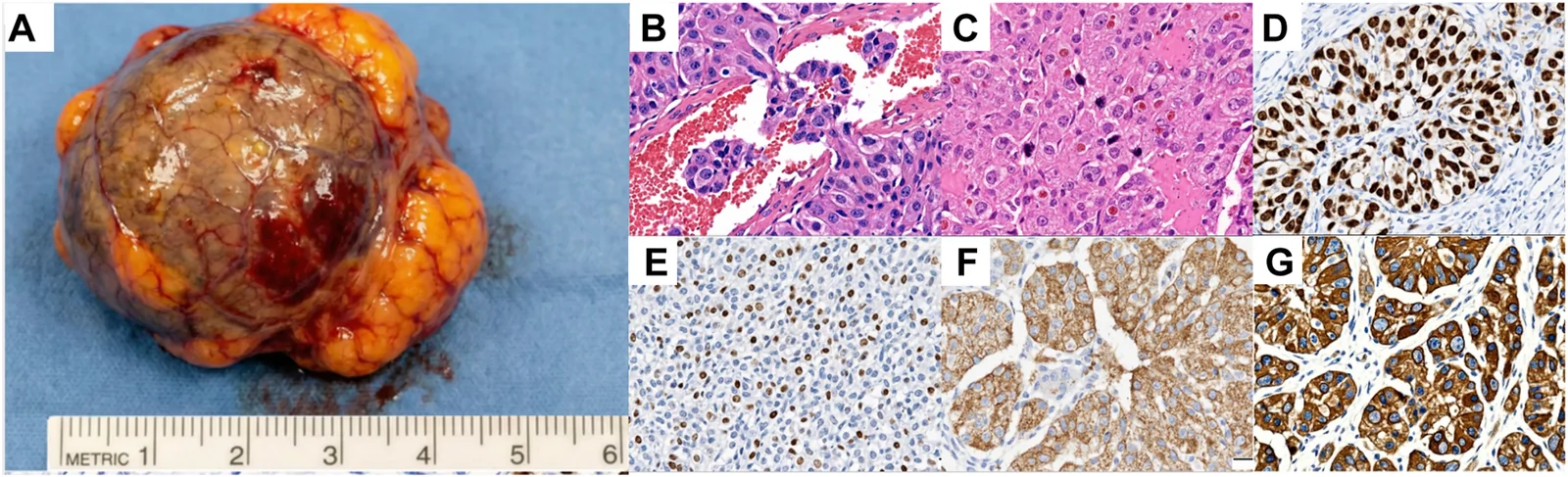
Pathologic findings of the adrenocortical carcinoma. (A) Gross pathology of the resected *left* adrenal gland showing a well-encapsulated, lobulated tumor measuring approximately 4.5 cm in greatest dimension. The intact specimen demonstrates a tan-gray capsular surface with prominent surface vasculature, surrounded by orange-yellow periadrenal adipose tissue, and a focal area of subcapsular hemorrhage (metric ruler shown for scale). (B) Histopathology demonstrating vascular invasion with tumor cells within a blood vessel lumen (H&E, ×400). (C) High-power view showing sheets of pleomorphic cells with high nuclear grade, prominent nucleoli, and abundant eosinophilic cytoplasm (H&E, ×400). (D) SF-1 immunohistochemistry revealing strong diffuse nuclear positivity, confirming adrenocortical origin (×200). (E) Ki-67 immunohistochemistry demonstrating a proliferation index of approximately 25% (×200). (F) Inhibin-alpha immunohistochemistry showing diffuse cytoplasmic positivity, supporting adrenocortical differentiation (×200). (G) Aldosterone synthase (CYP11B2) immunohistochemistry showing strong cytoplasmic positivity, confirming the tumor as the source of aldosterone production (×200). H&E, hematoxylin and eosin; SF-1, steroidogenic factor-1; CYP11B2, aldosterone synthase.

Immunohistochemical staining was strongly positive for SF-1, with diffuse nuclear positivity confirming the tumor's adrenocortical origin (Fig. 2 *D*). The Ki-67 proliferation index was approximately 25%, signifying a high-grade tumor with significant recurrence risk (Fig. 2 *E*).^22^^,^^23^ Inhibin-alpha immunohistochemistry showed diffuse cytoplasmic positivity, further supporting adrenocortical differentiation (Fig. 2 *F*). Aldosterone synthase (CYP11B2) immunostaining was also positive, with strong cytoplasmic staining confirming the tumor as the source of aldosterone production (Fig. 2 *G*). The tumor was completely excised with negative surgical margins, and no lymph node metastases were found, resulting in a final designation of ENSAT Stage II (T2*N*0M0).^1^

In the postoperative period, the patient required hydrocortisone replacement for iatrogenic adrenal insufficiency, a consequence of chronic HPA axis suppression.^1^ Her serum potassium normalized without supplementation. Her hypertension and diabetes improved dramatically, permitting a significant reduction in her medication burden. Following a multidisciplinary tumor board discussion that considered the tumor's high-risk features (hormone secretion and Ki-67 > 10%), adjuvant treatment with mitotane was recommended per ESE-ENSAT guidelines.^1^^,^^23^ Mitotane was initiated 6 weeks after surgery and titrated to a therapeutic level (14-20 μg/mL) over 3 months, with a corresponding increase in her hydrocortisone dose to counteract mitotane's metabolic effects.^24^ Follow-up surveillance with imaging and hormonal assessments is conducted every 3 months. At the 6-month follow-up, there was no evidence of disease recurrence, and hormonal assessments confirmed ongoing remission. This surveillance will continue every 3-6 months for the first 2 years, then annually for at least 5 years, in accordance with current guidelines.^1^ Postoperative aldosterone was suppressed at <4 ng/dL with a corresponding plasma renin activity of 2.5 ng/mL/h, confirming biochemical remission.

## Discussion

This report describes an unusual case of ACC defined by the co-secretion of cortisol and aldosterone.^4^^,^^5^ The patient's clinical picture, dominated by severe hypertension, diabetes, and hypokalemia, alongside classic Cushingoid features, guided the comprehensive workup that ultimately revealed the tumor's dual functionality.^4^ The diagnosis of ACC was solidified by the combination of imaging findings, a large tumor size, and a definitive Weiss score of 6 on histopathology.^21^

The genetic basis for such dual secretion is thought to involve a functional overlap between the genes encoding 11β-hydroxylase and aldosterone synthase on chromosome 8.^4^ While molecular testing for mutations in genes such as TP53, CTNNB1, and IGF2, which are commonly altered in ACC, was not performed in this case due to logistical constraints, these alterations are known to contribute to disordered steroidogenesis and could underlie the dual-hormone secretion observed.^25^ While this phenomenon has been described more frequently in benign adenomas, its occurrence in the context of carcinoma is rare.^4^^,^^5^ A comprehensive literature review identified only 14 prior cases of dual aldosterone-cortisol secreting ACC reported between 2004 and 2025 (Table 1).^5^, ^6^, ^7^, ^8^, ^9^, ^10^, ^11^ These cases demonstrate a female predominance (73%), a wide age range (18-77 years), and variable tumor sizes (3.5-23.0 cm). Notably, some cases exhibited triple hormone secretion, with concurrent androgen, estradiol, or catecholamine production.^7^^,^^10^ The elevated normetanephrine in one case^10^—which presented with elevated plasma normetanephrine but normal metanephrine—was not associated with histologic features of pheochromocytoma. This suggests the finding may represent aberrant tumor metabolism rather than true catecholamine co-secretion, highlighting that biochemical findings do not always align with histology in ACC. The biochemical evaluation of primary aldosteronism in the presence of Cushing's syndrome may differ from those without hypercortisolism, as one would expect lower aldosterone levels secondary to the binding of excess cortisol to the mineralocorticoid receptor. In a study of 17 patients with Cushing disease, the mean aldosterone level was 3.7 ng/dL.^26^ This underscores the significance of the aldosterone level in our patient. Furthermore, while a significant portion of patients with primary aldosteronism may have some degree of autonomous cortisol secretion, particularly those with larger adenomas, the excess cortisol secretion in ACC patients with PA has not been well studied.^27^

Effective management of ACC necessitates a coordinated, multidisciplinary team.^1^ Radical surgical resection remains the primary modality for localized disease.^1^ In this case, successful resection was preceded by crucial preoperative stabilization, including the correction of life-threatening hypokalemia.^19^ The decision to proceed with adjuvant mitotane was driven by established high-risk features, including the elevated Ki-67 index.^1^^,^^23^ Adjuvant mitotane has been demonstrated to improve recurrence-free survival in patients who have undergone complete resection of their ACC.^24^

A thorough preoperative endocrine assessment is paramount.^1^^,^^18^ Identifying cortisol excess is essential for the anticipation and management of postoperative adrenal insufficiency, a potentially fatal complication.^1^ Likewise, diagnosing hyperaldosteronism is critical for managing perioperative hypertension and electrolyte disturbances, thereby minimizing cardiovascular risks such as cardiac arrhythmias.^19^ The 6-week delay in initiating mitotane in this case, driven by the need to confirm primary aldosteronism off interfering medications, highlights a clinical challenge. While this delay is unlikely to have significantly impacted the outcome given the aggressive nature of ACC, it underscores the importance of prompt diagnosis and treatment. The presence of vascular invasion at diagnosis suggests that tumor aggressiveness was the primary driver of prognosis.

The prognosis for ACC is generally poor, with 5-year survival rates for Stage II disease around 50% to 65%.^1^ Prognostic indicators include tumor stage, resection status, Ki-67 index, and hormonal activity.^23^ A Ki-67 index above 10% is a well-established marker of aggressive behavior and a higher likelihood of recurrence.^23^ Our patient's index of 25% places her in this high-risk group. Furthermore, cortisol-secreting tumors are associated with a worse prognosis compared to nonfunctioning tumors, an effect potentially mediated by the metabolic and immunosuppressive consequences of hypercortisolism.^28^ The prognostic significance of aldosterone co-secretion is not well-established due to its rarity.^3^^,^^4^ The withdrawal of interfering medications for the diagnosis of primary aldosteronism delayed surgical intervention by approximately 6 weeks. While it is difficult to ascertain whether this delay contributed to vascular invasion, the aggressive nature of ACC, with a high Ki-67 index, suggests that the potential for invasion was already high. The natural history of ACC is variable, but rapid growth and invasion are hallmarks of the disease.^1^ Accordingly, vigilant surveillance with imaging and hormone monitoring (including serum cortisol, aldosterone, and renin) every 3 months for the first 2 years is warranted to detect recurrence early.^1^

## Conclusion

This case of a dual cortisol- and aldosterone-secreting ACC illustrates the critical importance of a comprehensive hormonal workup in patients with adrenal tumors, regardless of the dominant clinical syndrome.^1^^,^^4^ Dual hypersecretion is a rare but significant clinical entity that demands a high degree of clinical suspicion and a multidisciplinary management approach to optimize patient outcomes in this aggressive disease.^1^

## Statement of Patient Consent

The authors affirm that the patient provided written informed consent for the publication of this case report and any accompanying images.

## Ethics Statement

This case report was conducted in accordance with the Declaration of Helsinki. Written informed consent was obtained from the patient for publication of this case report and accompanying images.

## Disclosure

The author has no conflict of interest to disclose.
